# Supplementary material for: In Vivo and In Vitro Enhanced Tumoricidal Effects of Metformin, Active Vitamin D3, and 5-Fluorouracil Triple Therapy against Colon Cancer by Modulating the PI3K/Akt/PTEN/mTOR Network
Source: Cancers (Basel). 2022 Mar 17;14(6):1538. doi: 10.3390/cancers14061538 (PMC8946120; doi:10.3390/cancers14061538)

Detection of CCND1 protein (34 KD) by WB

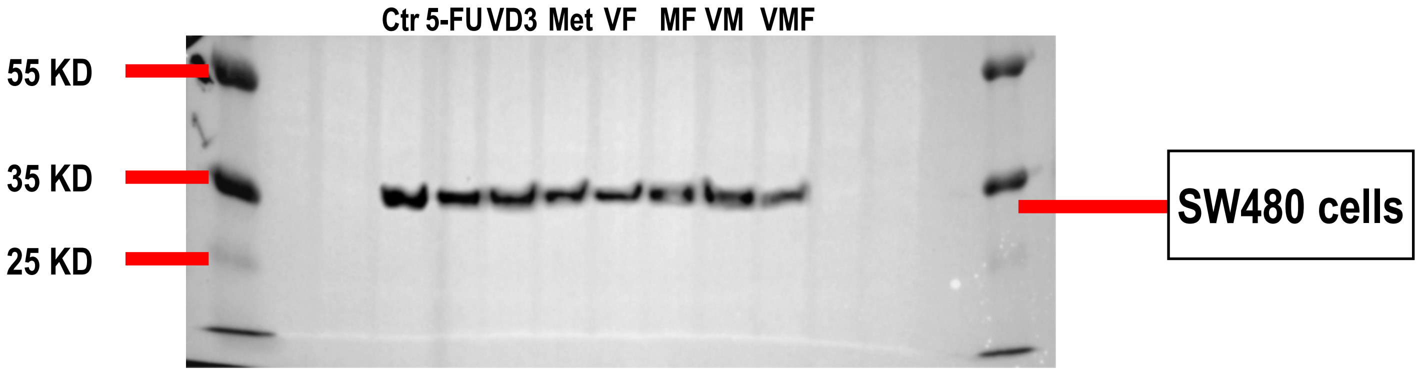

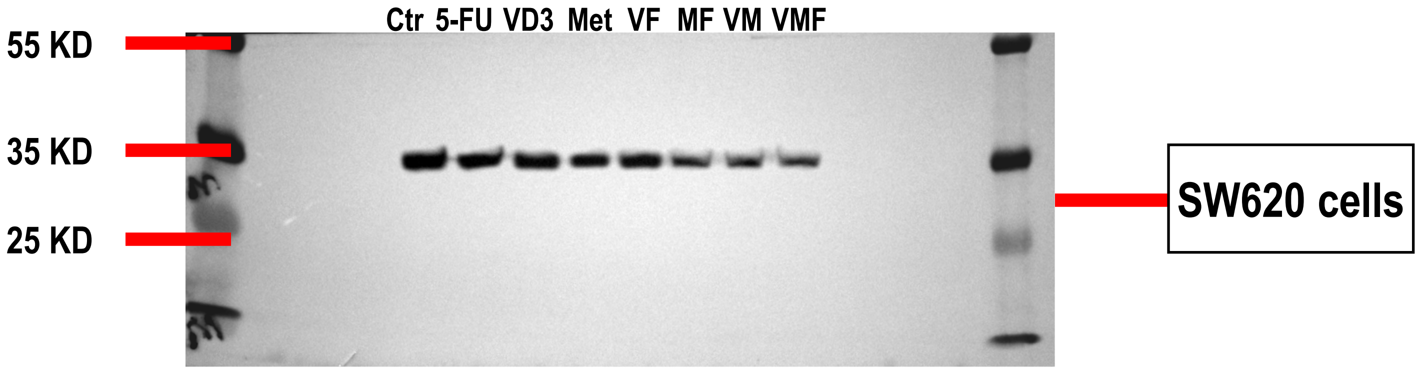

Detection of CCND3 protein (31 KD) By WB

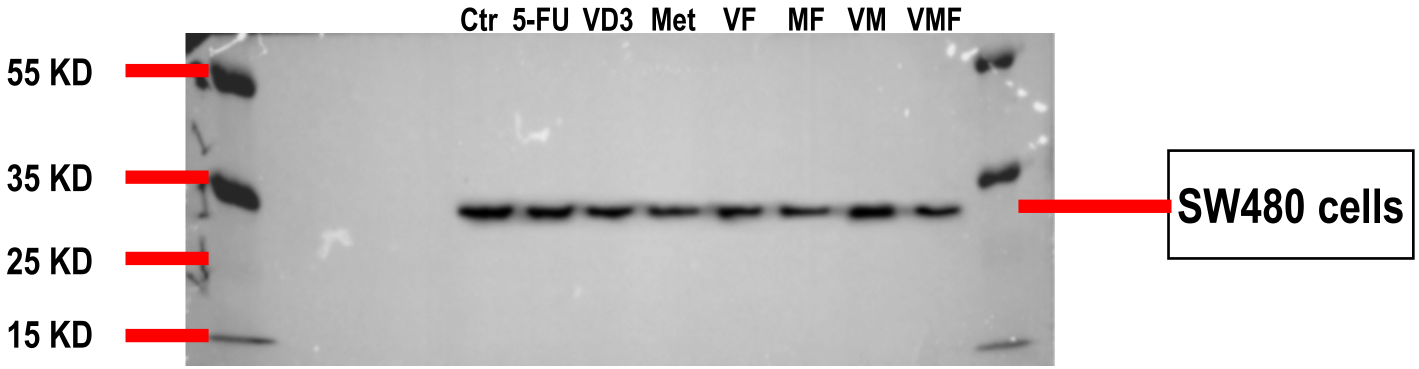

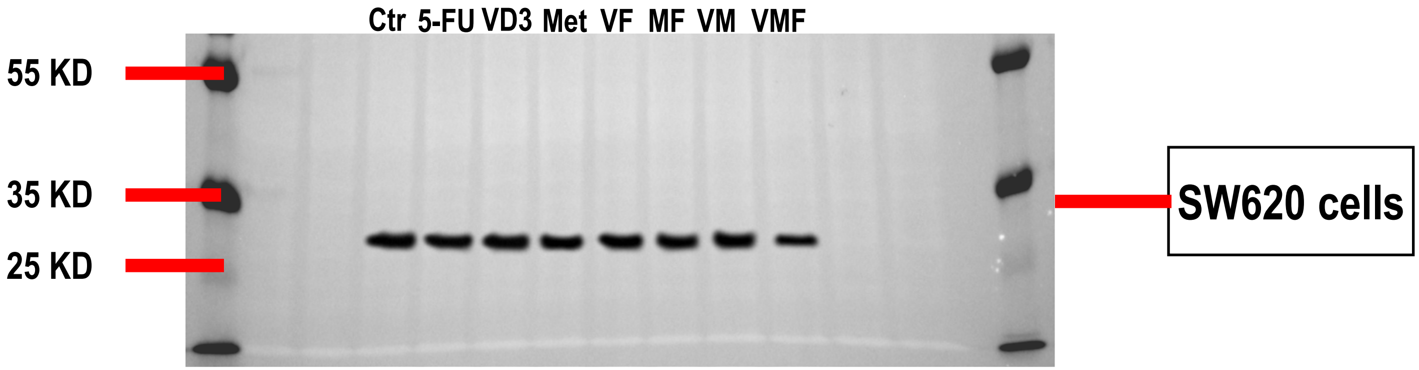

Detection of p21 protein (21 KD) by WB

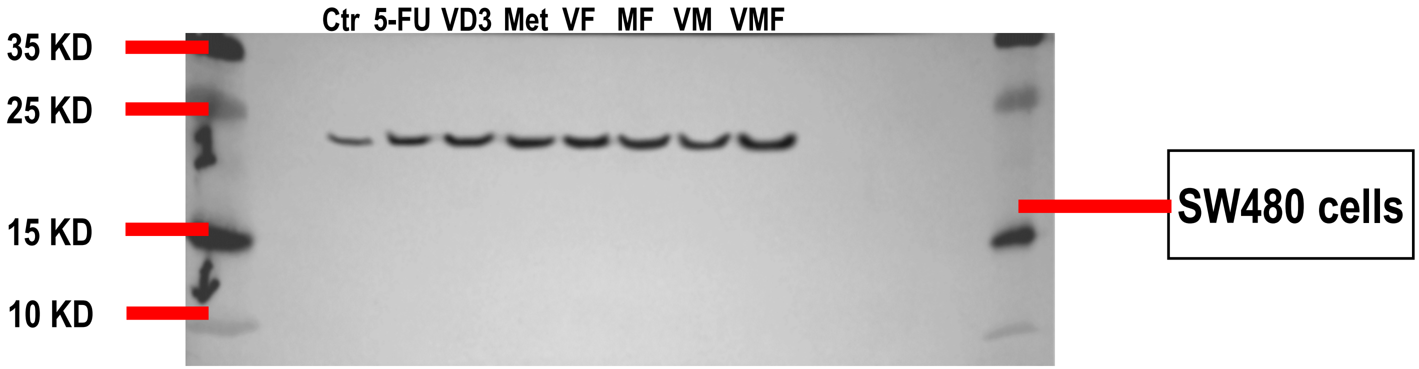

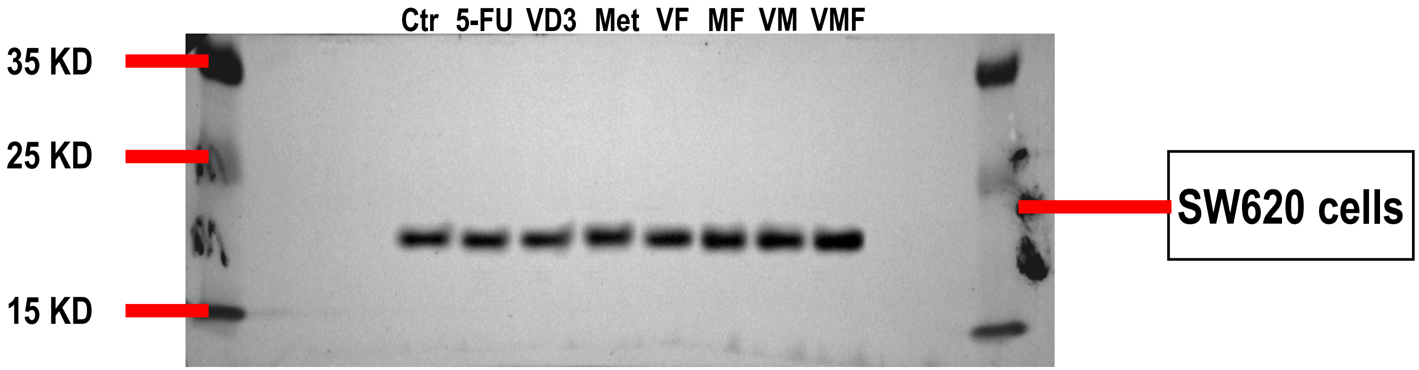

Detection of p27 protein (27 KD) by WB

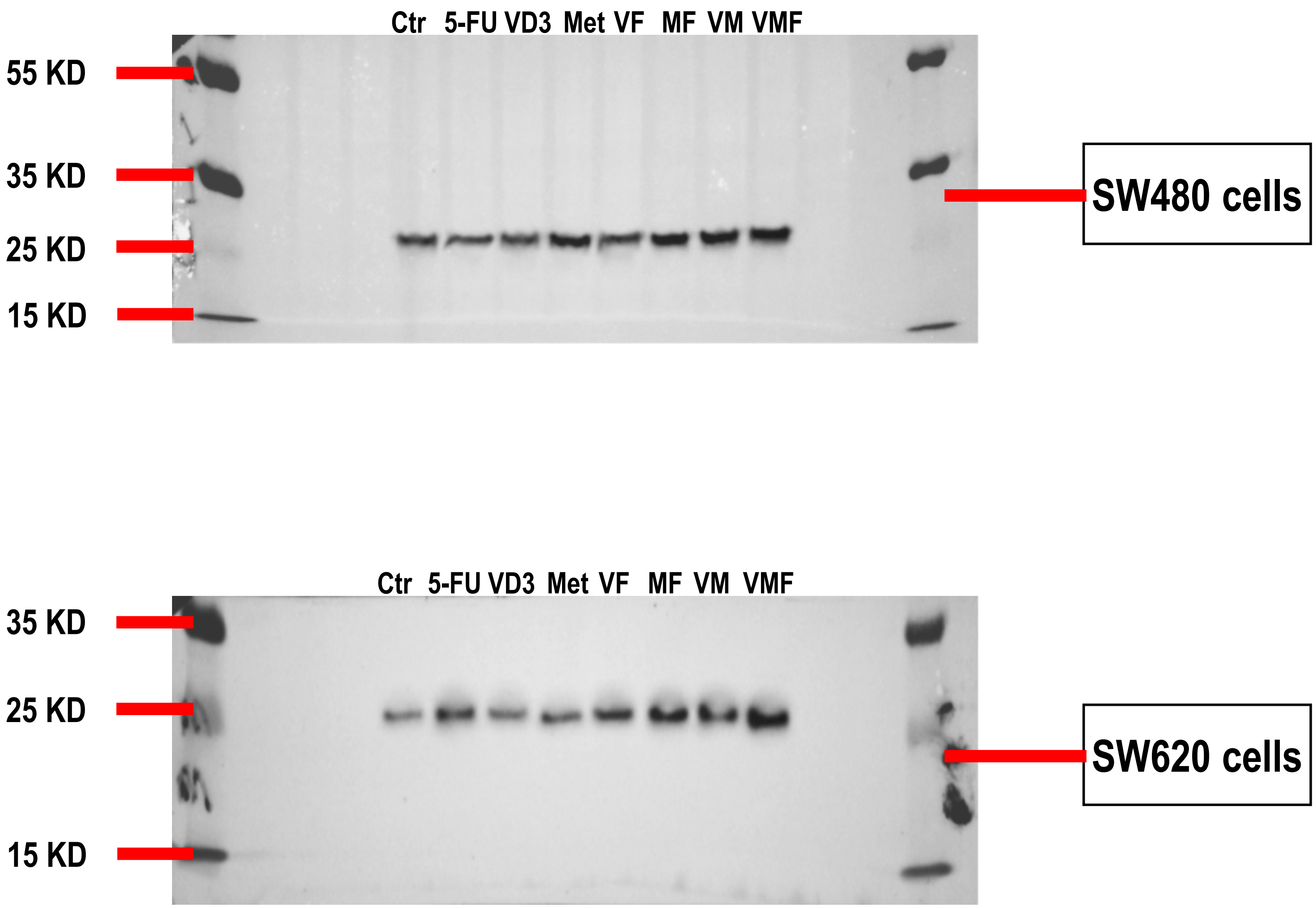

Detection of BCL2 protein (26 KD) by WB

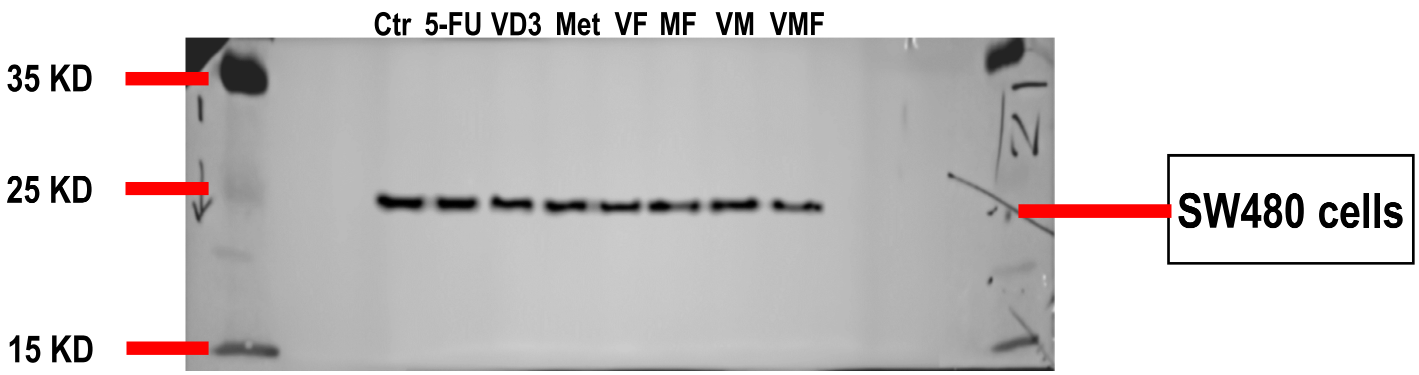

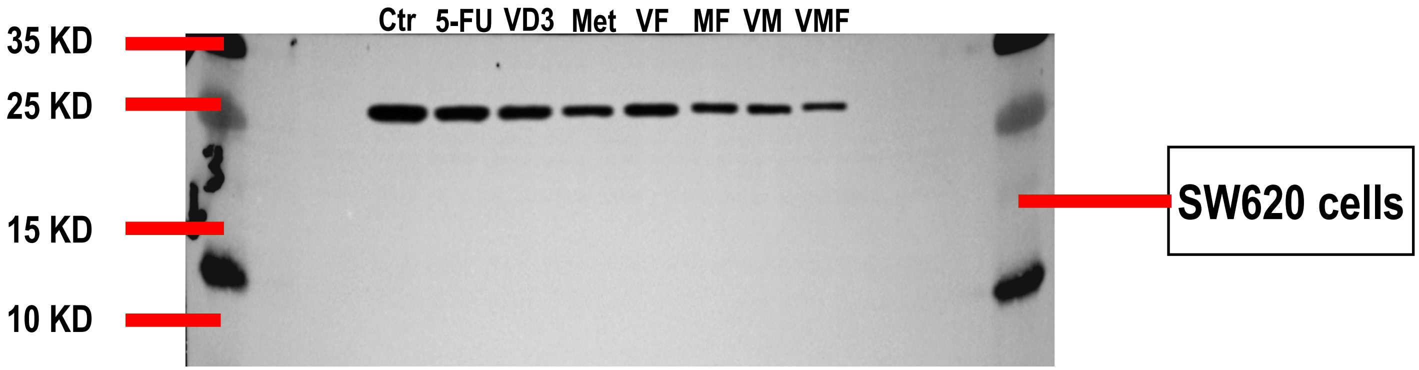

Detection of BAX protein (20 KD) by WB

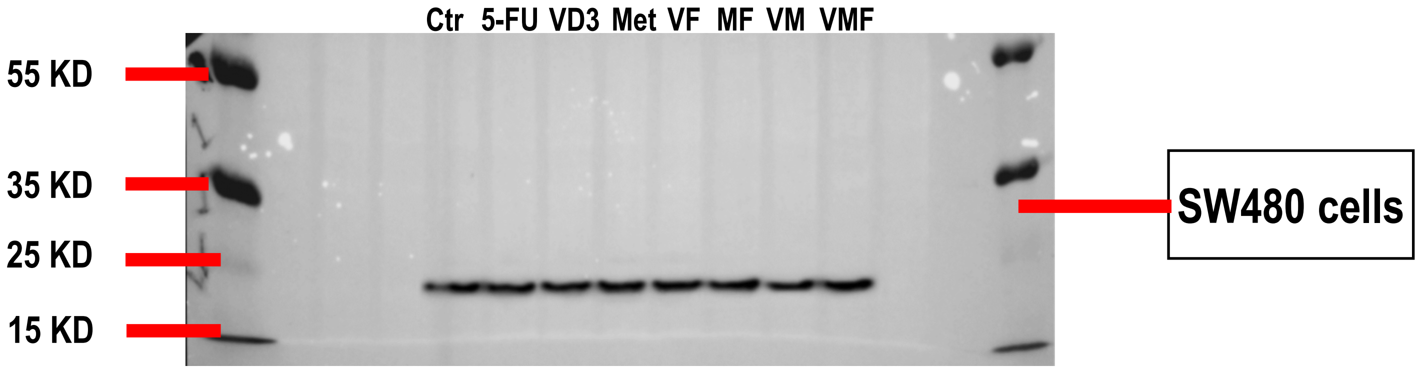

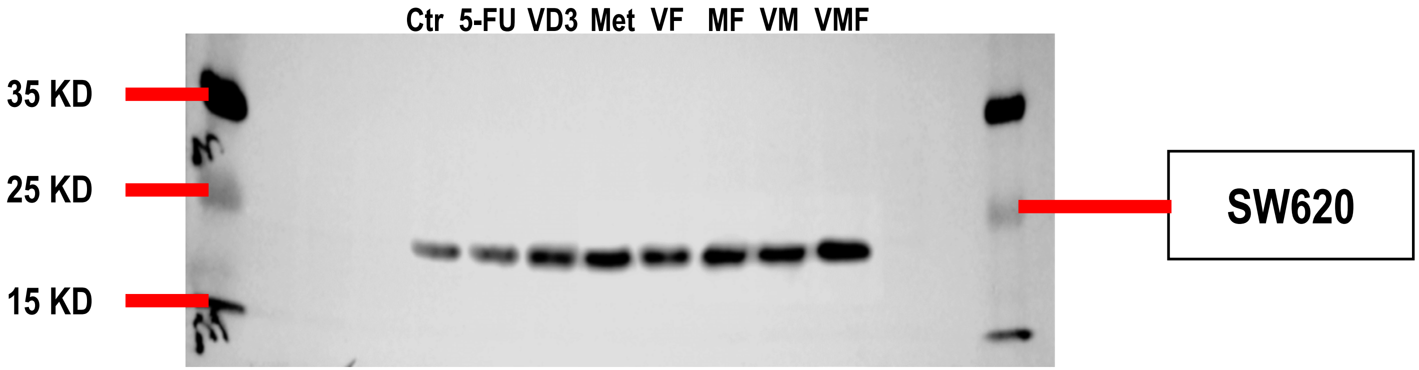

Detection of Cytochrome C protein (12 KD) by WB

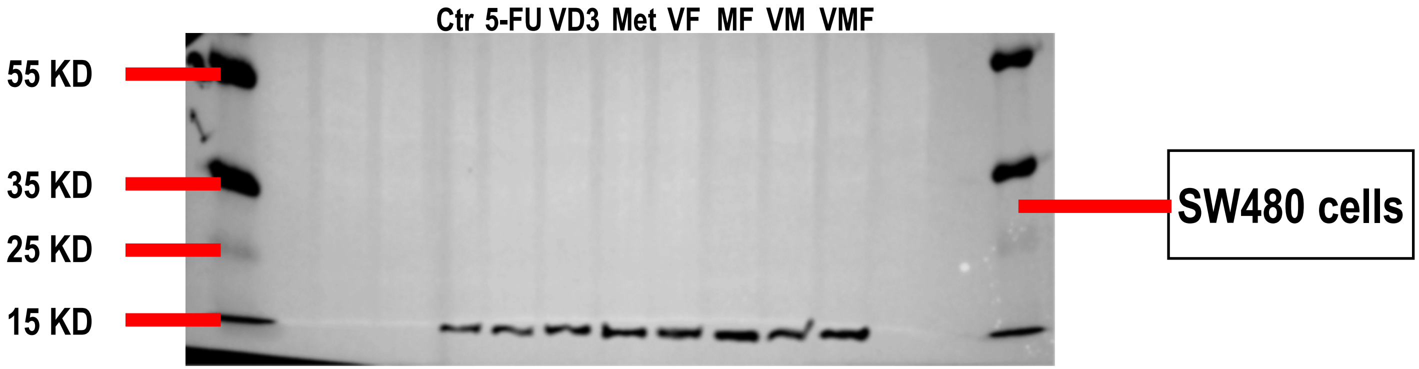

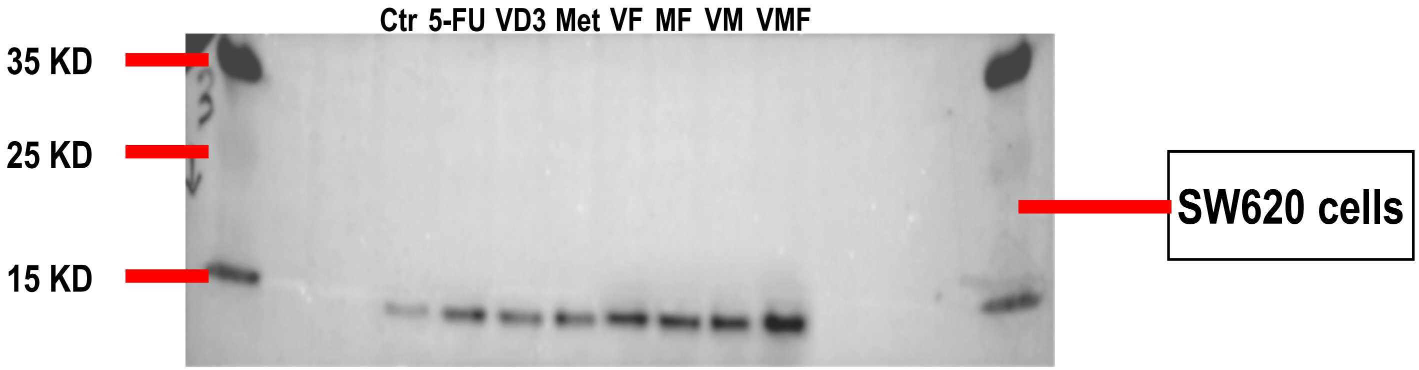

Detection of Cleaved Caspase-3 protein (17 KD) by WB

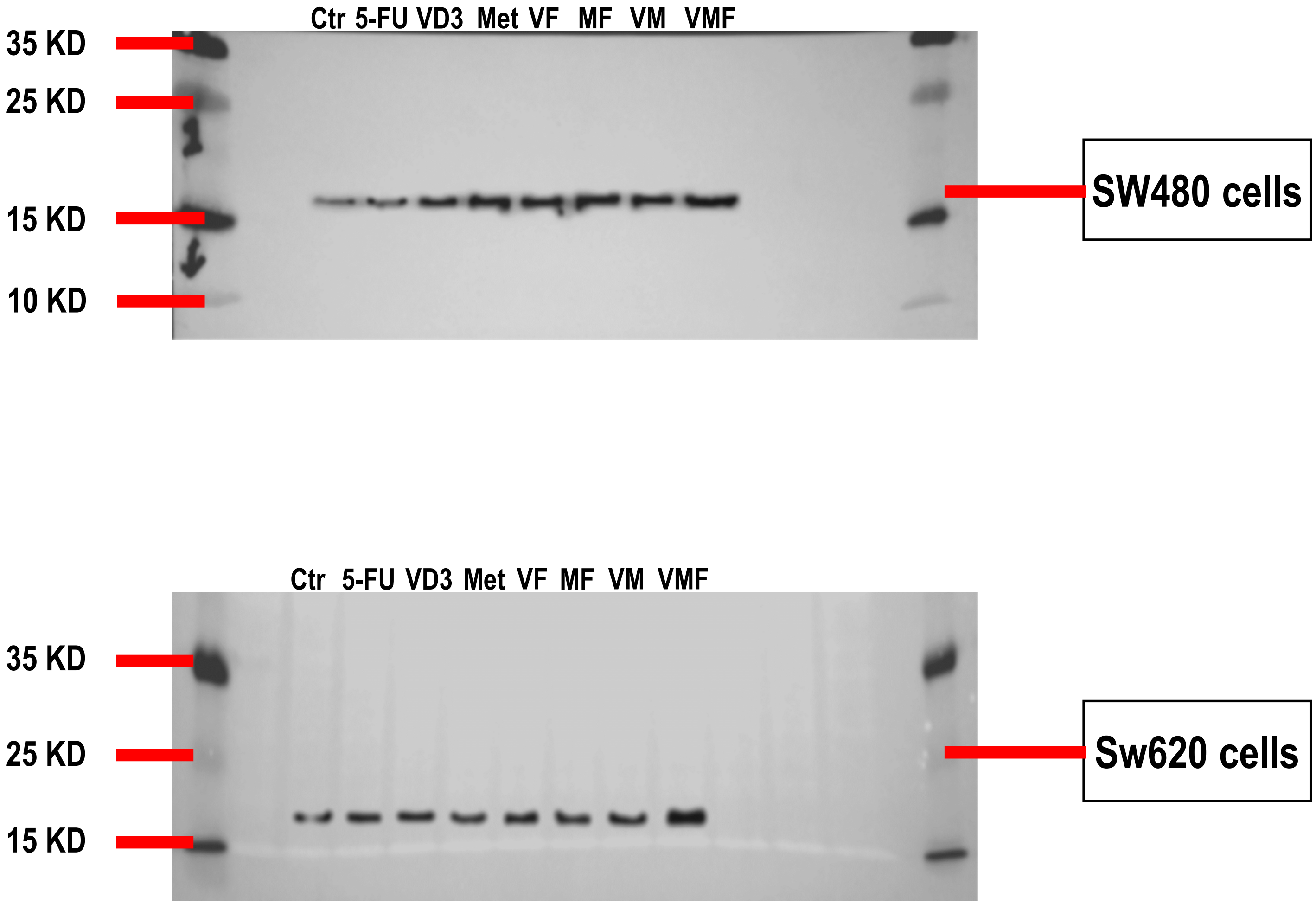

Detection of PI3K-p85α protein (85 KD) by WB

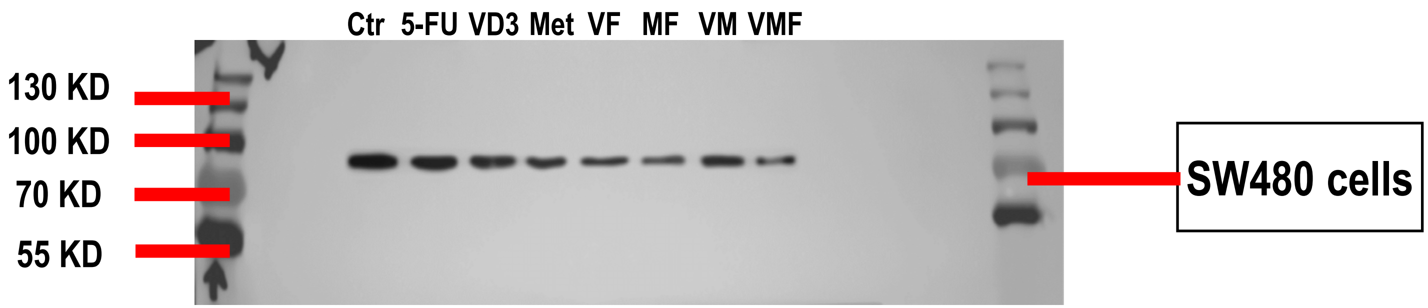

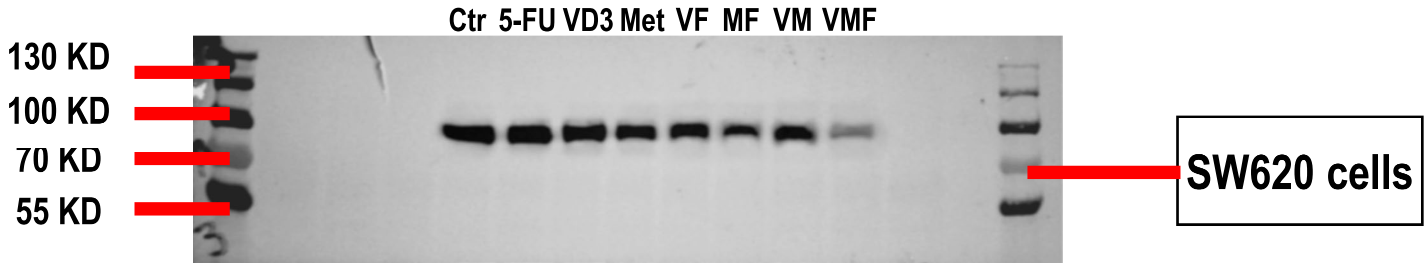

Detection of PTEN protein (54 KD) by WB

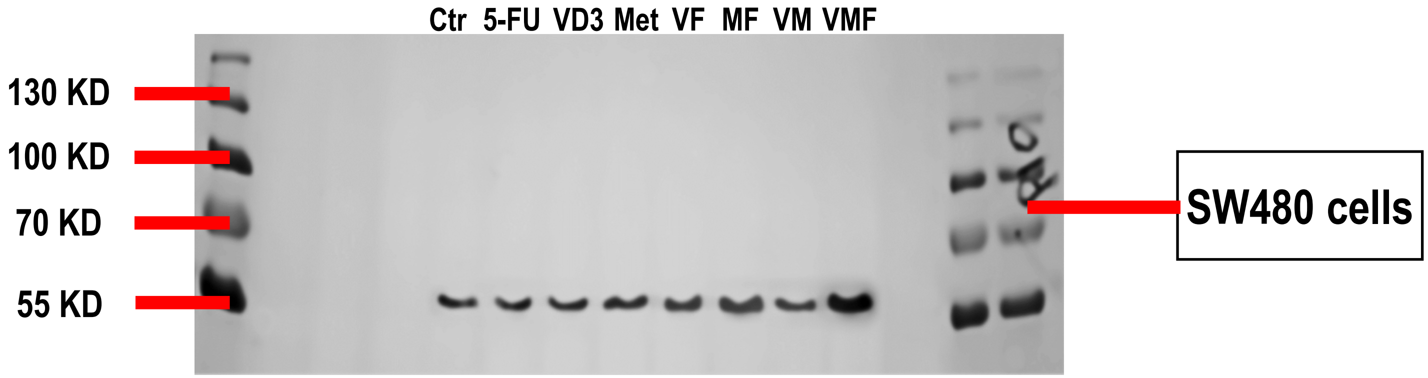

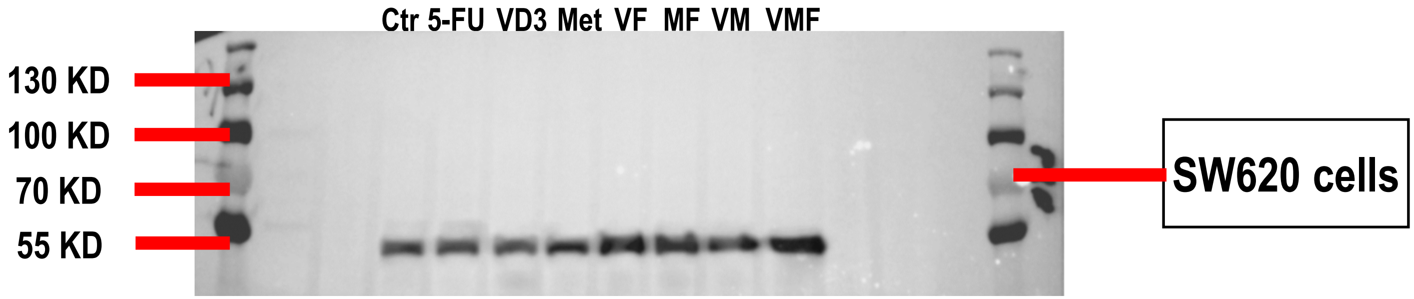

Detection of Akt1 protein (60 KD) by WB

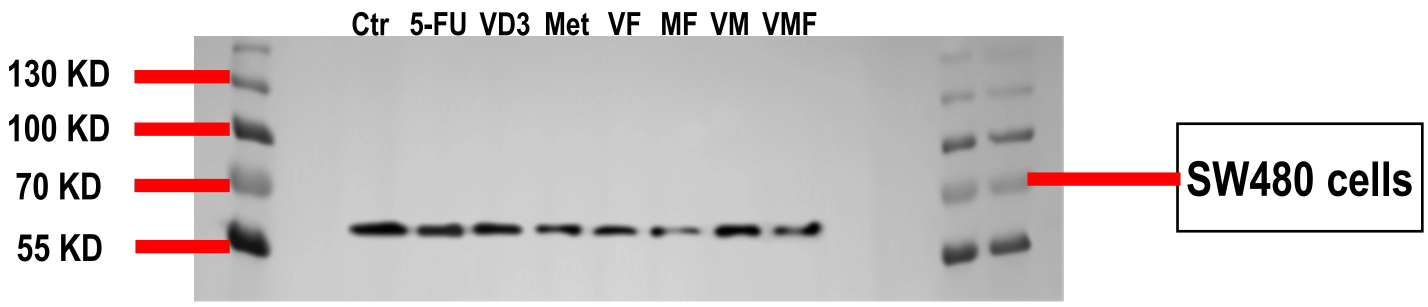

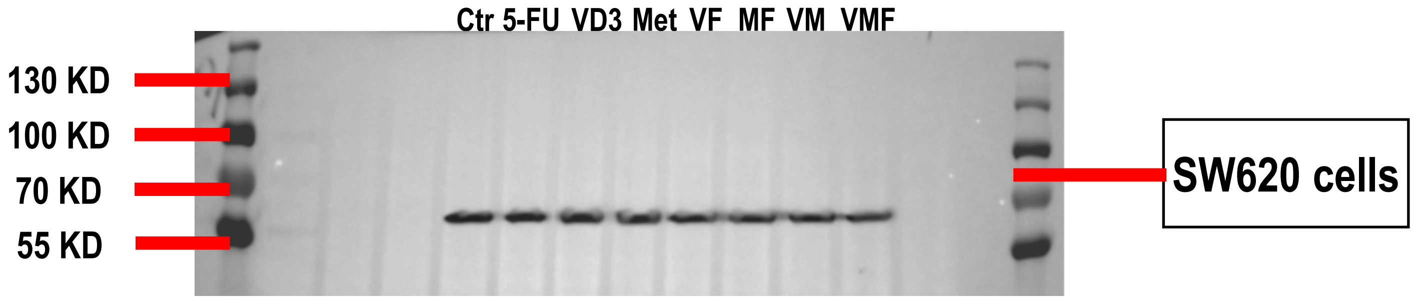

Detection of mTOR protein (289 KD) by WB

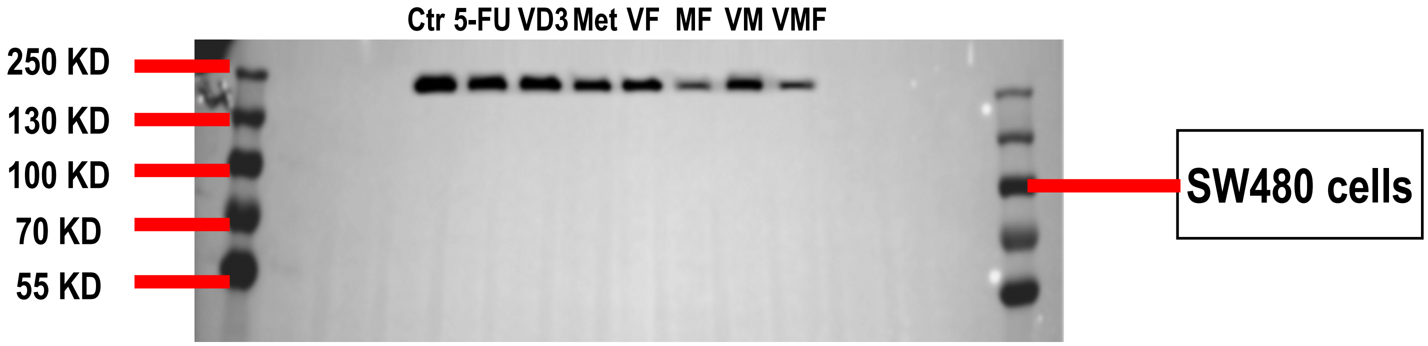

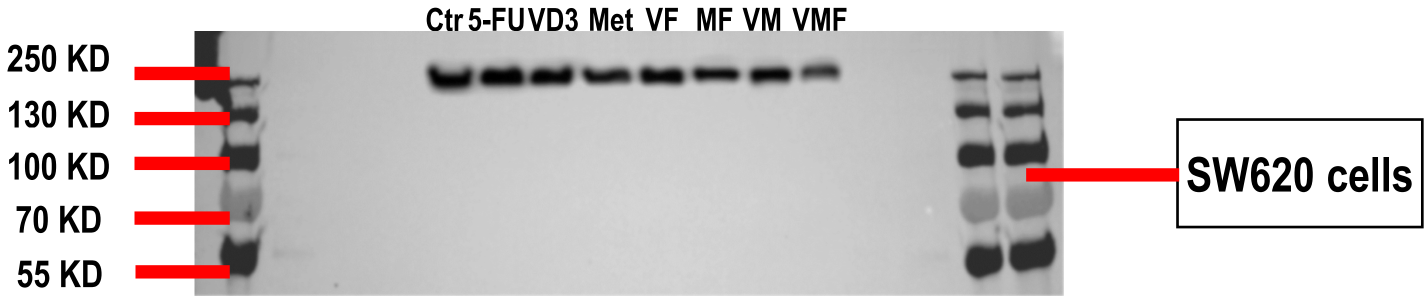

Detection of GAPDH protein (37 KD) by WB

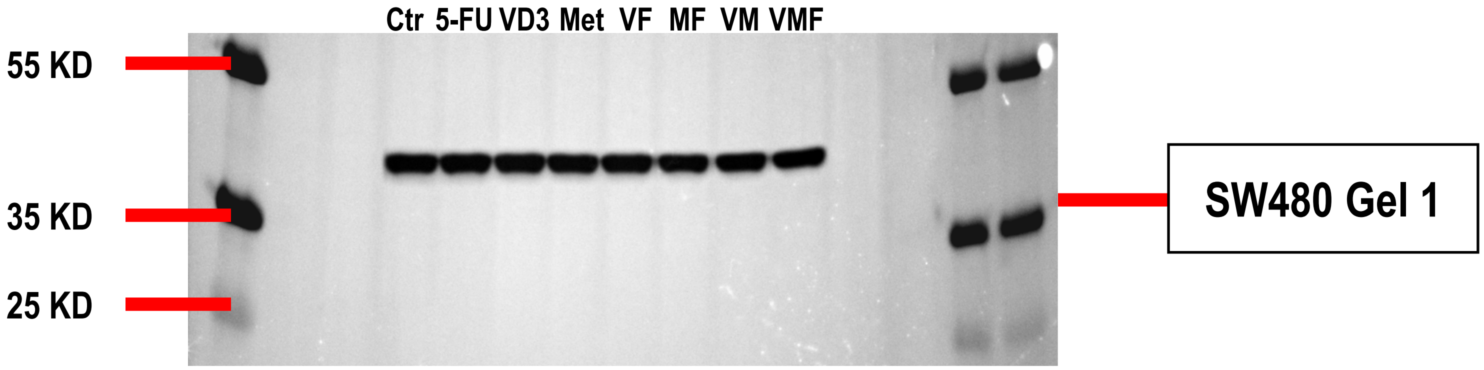

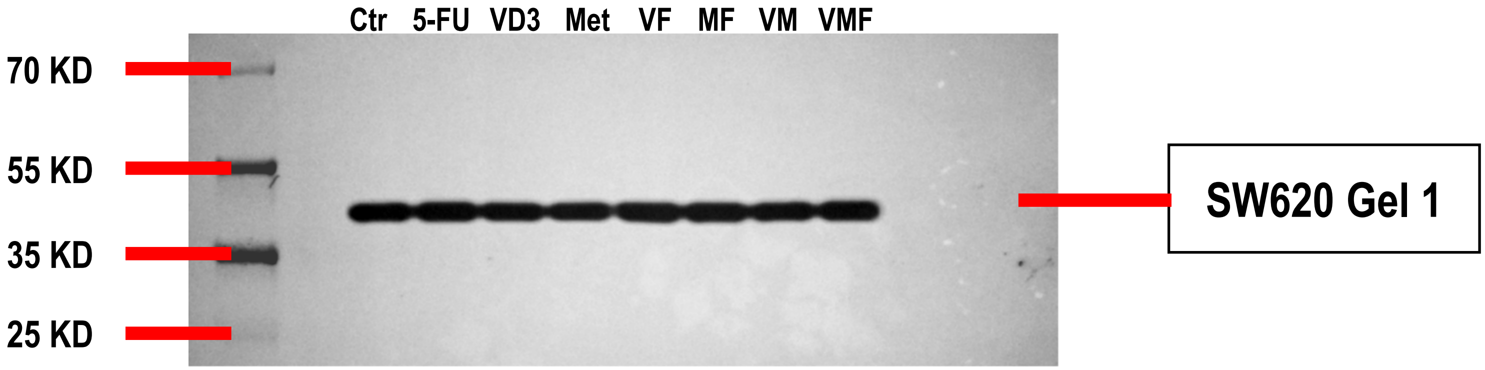

Detection of GAPD protein (37 KD) By WB

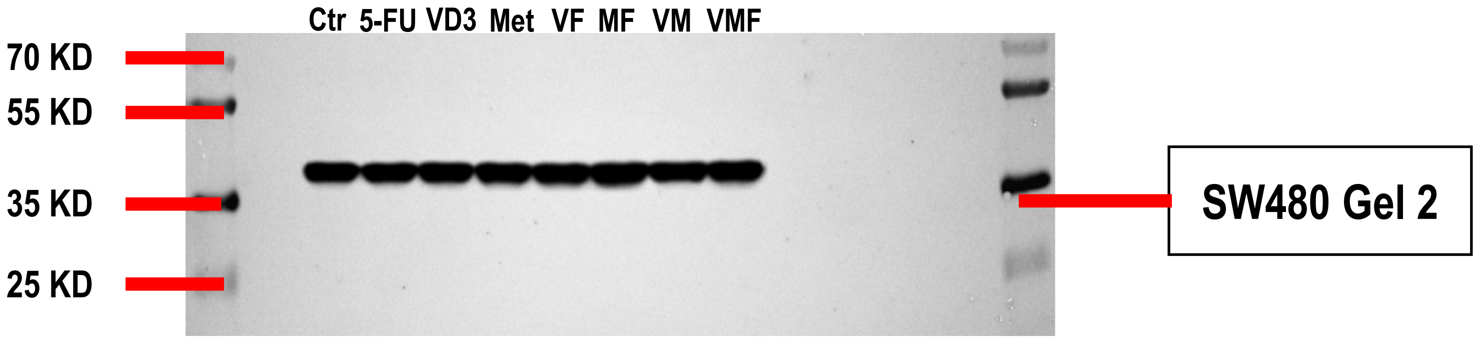

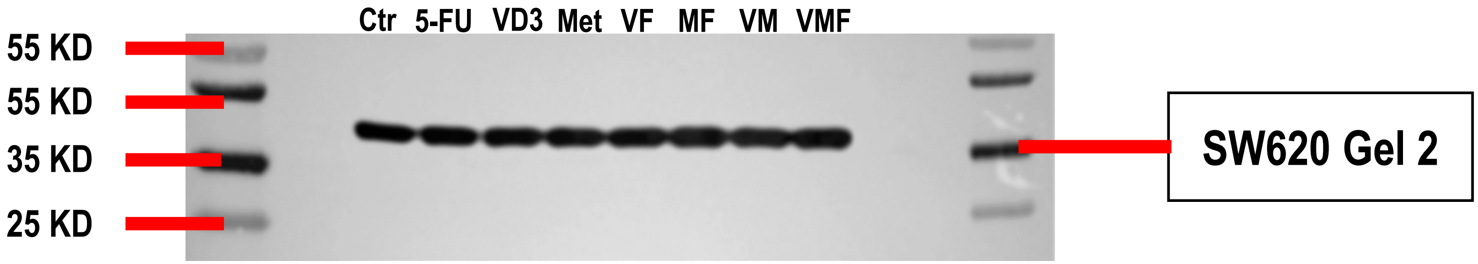

Detection of GAPDH protein (37 KD) by WB

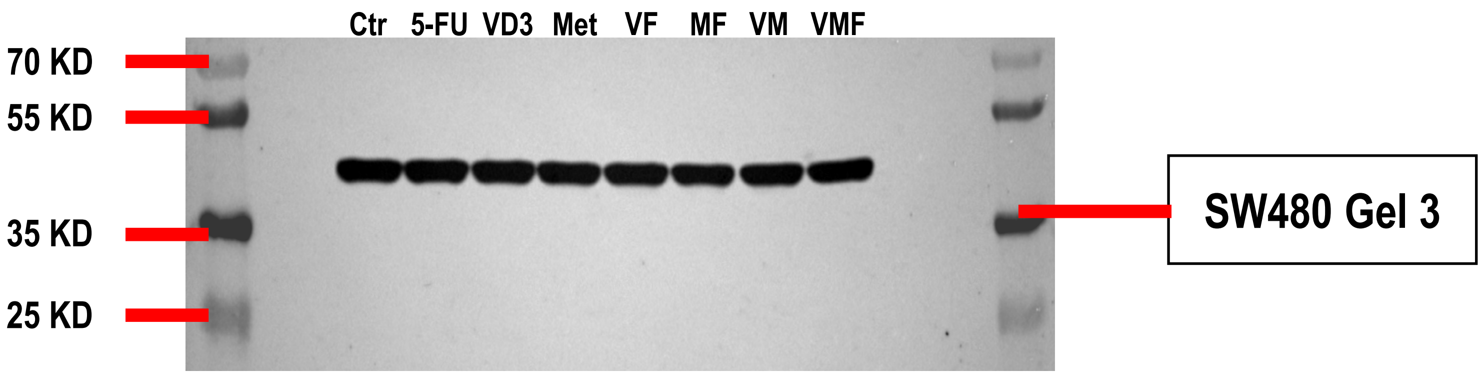

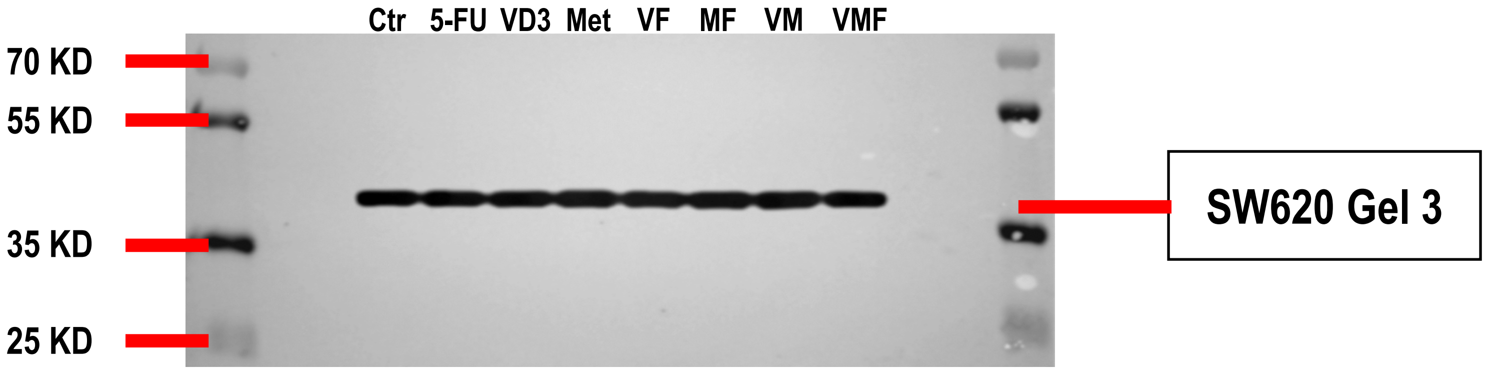

Detection of GAPDH protein (37 KD) by WB

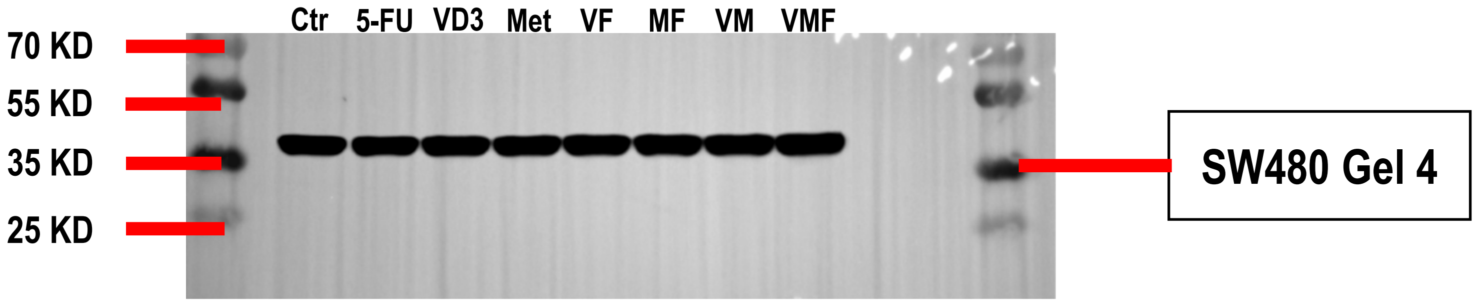

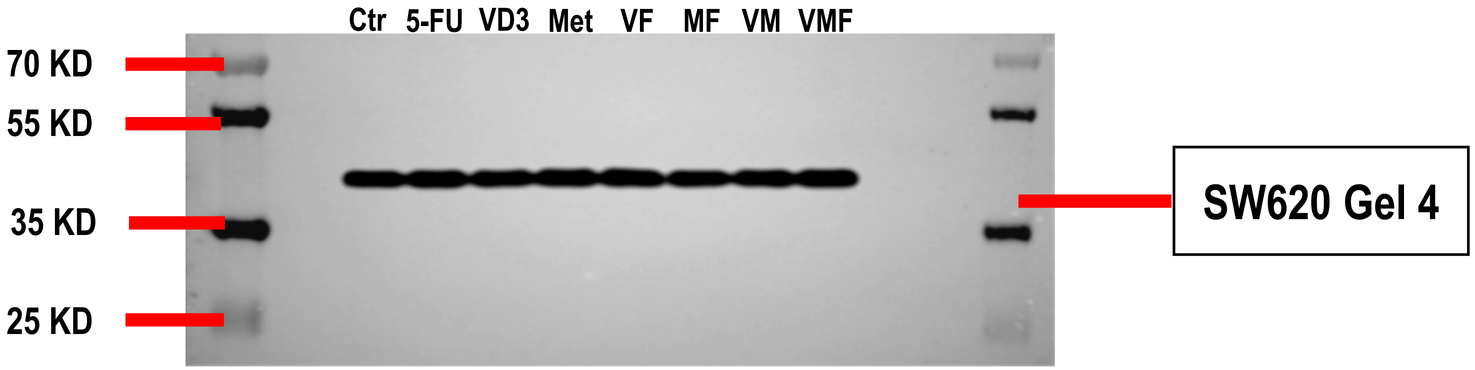

Detection of GAPDH protein (37 KD) by WB

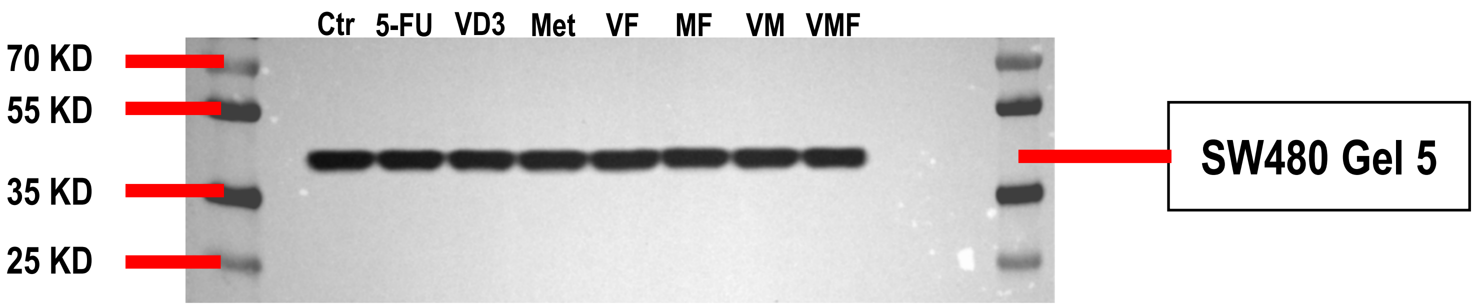

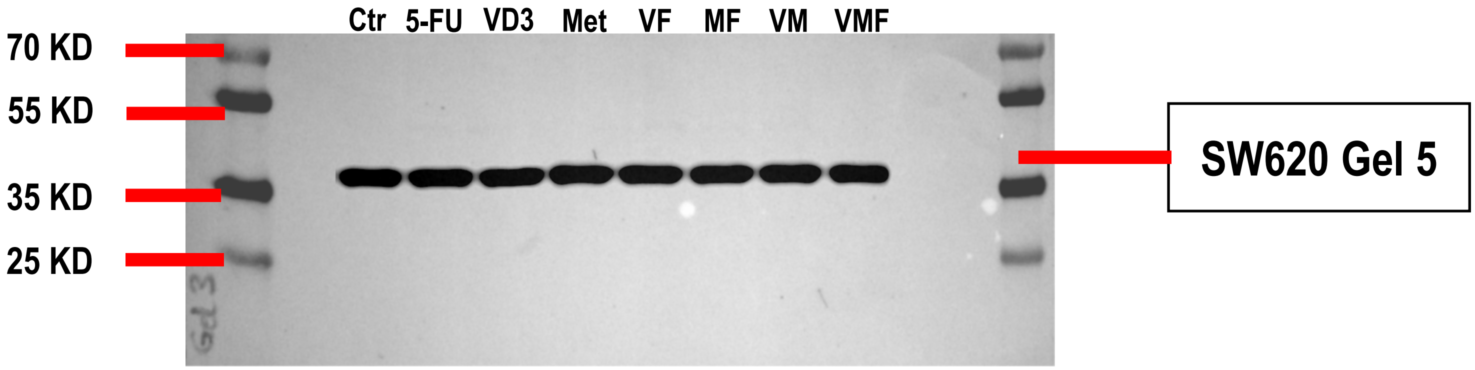

Detection of GAPDH protein (37 KD) by WB

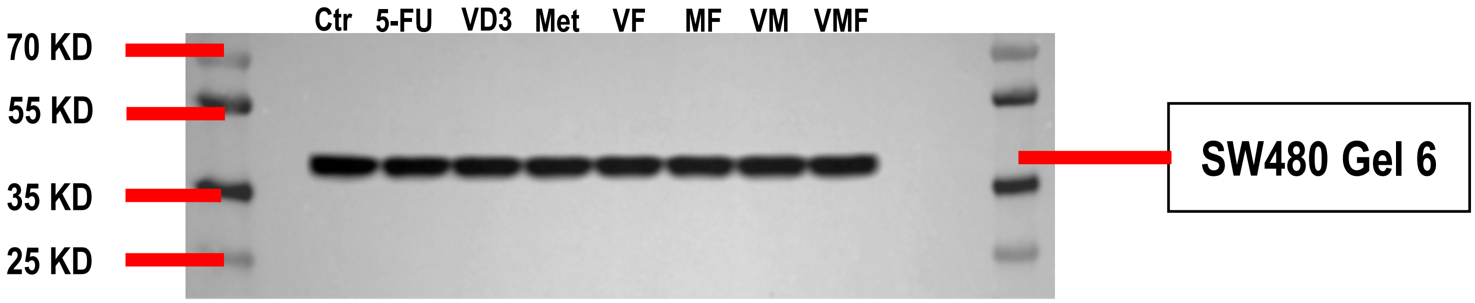

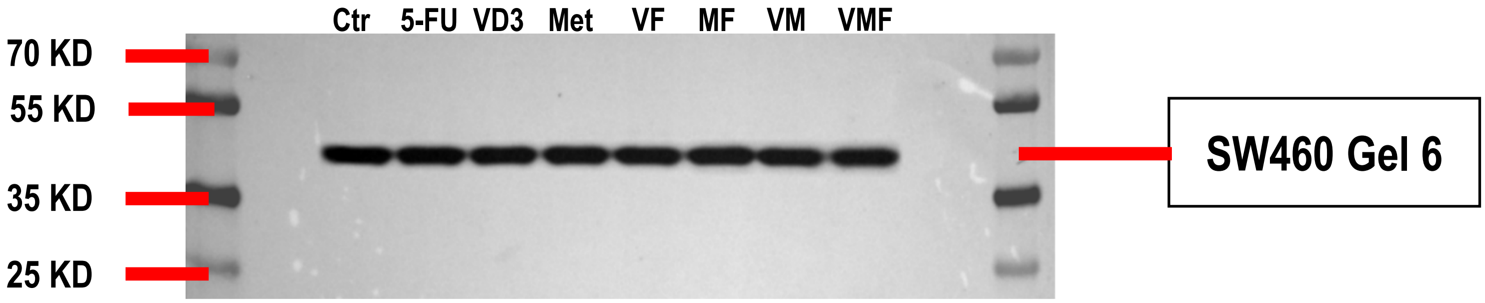

Detection of GAPDH protein (37 KD) by WB

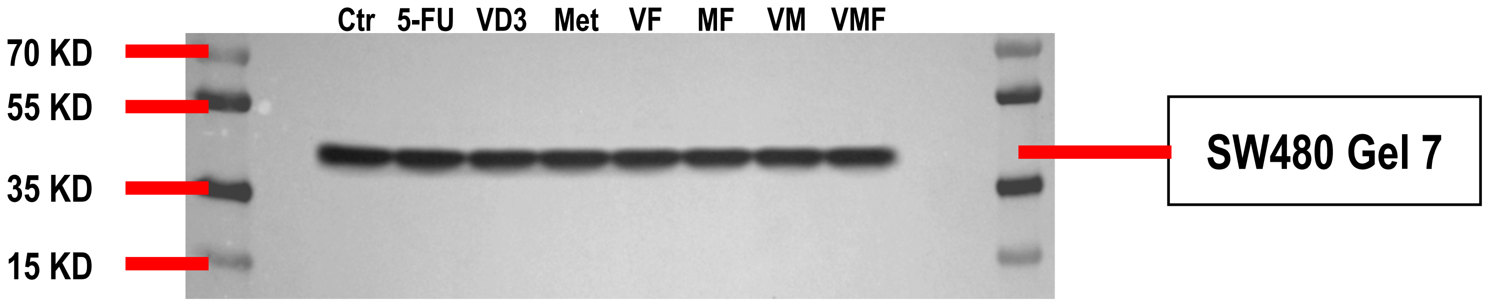

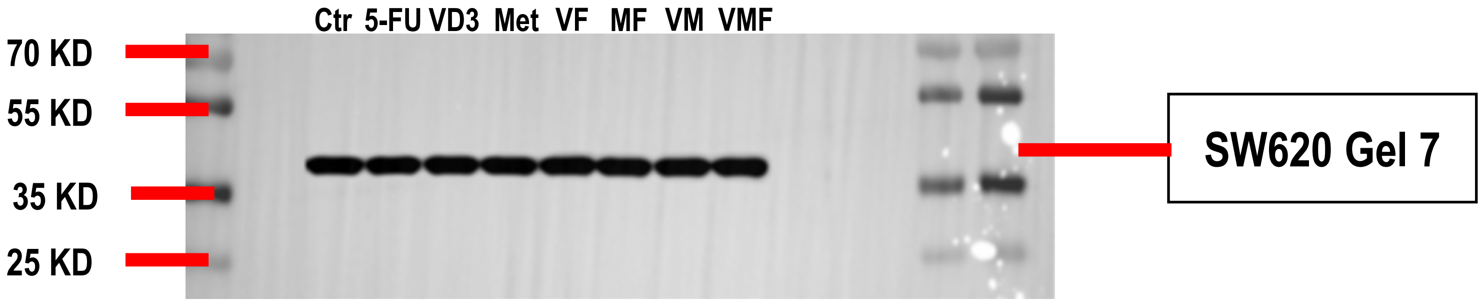

Detection of GAPDH protein (37 KD) by WB

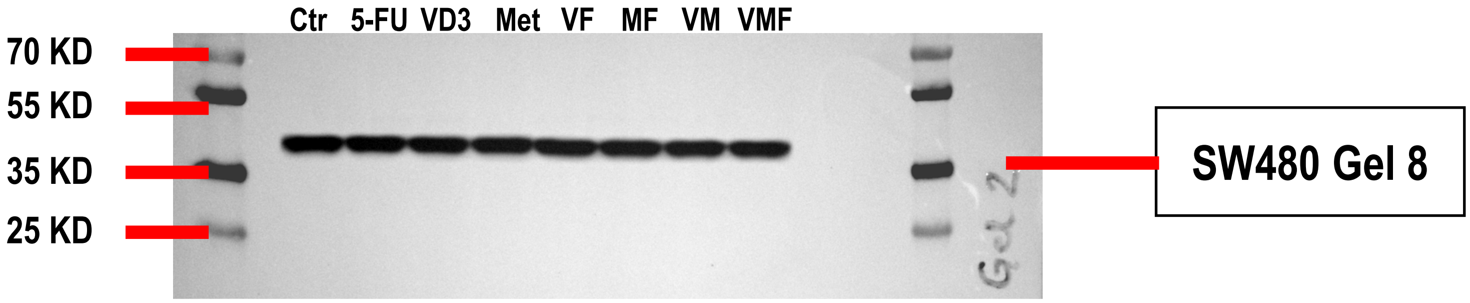

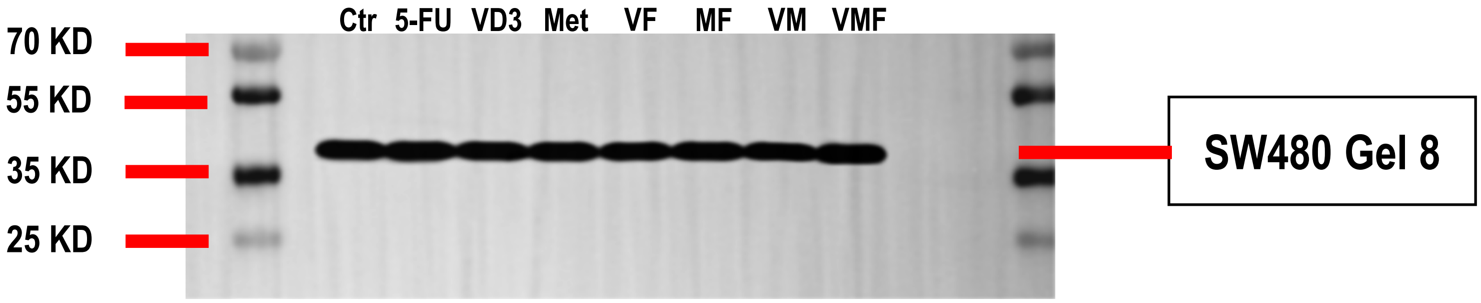

Detection of GAPDH protein (37 KD) by WB

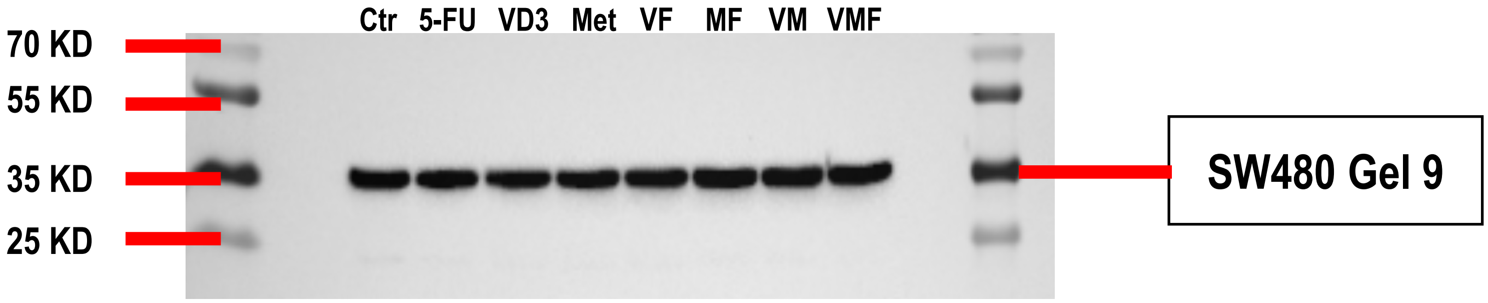

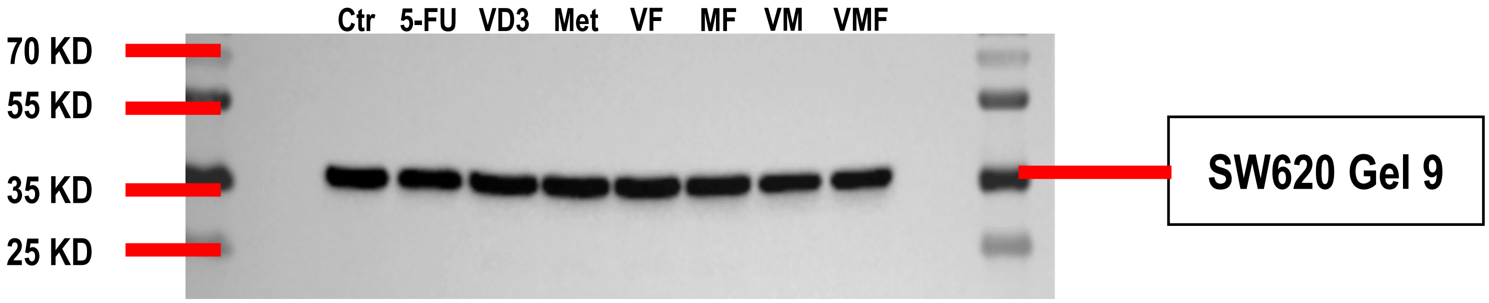

Detection of GAPDH protein (37 KD) by WB

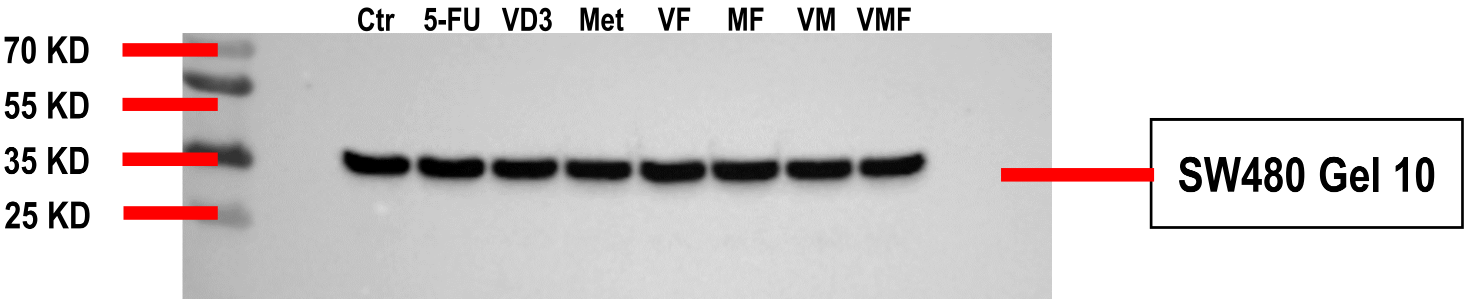

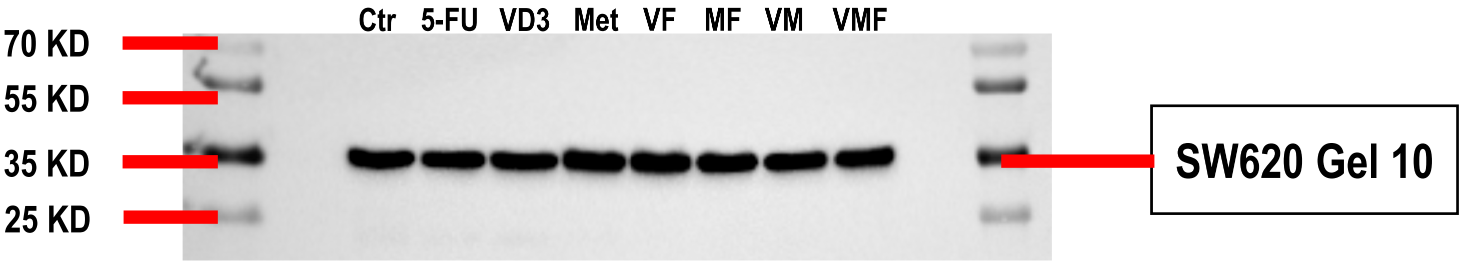

Detection of GAPDH protein (37 KD) by WB

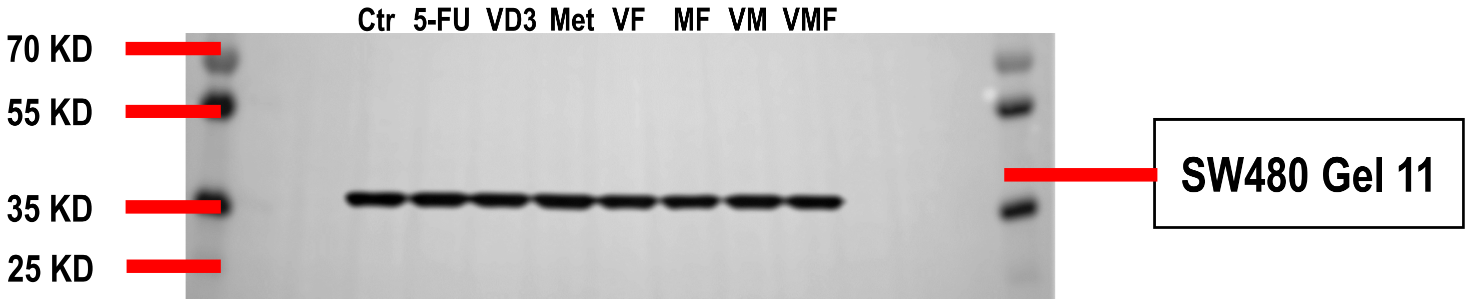

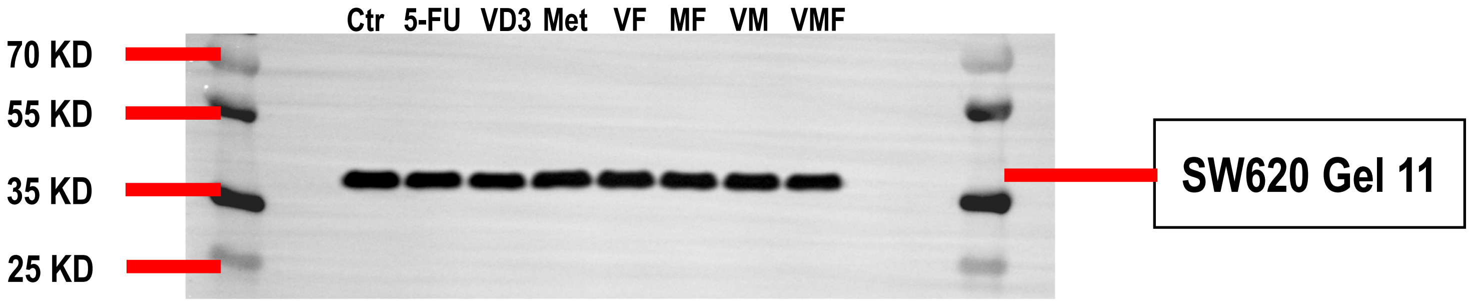

Detection of GAPDH protein (37 KD) by WB

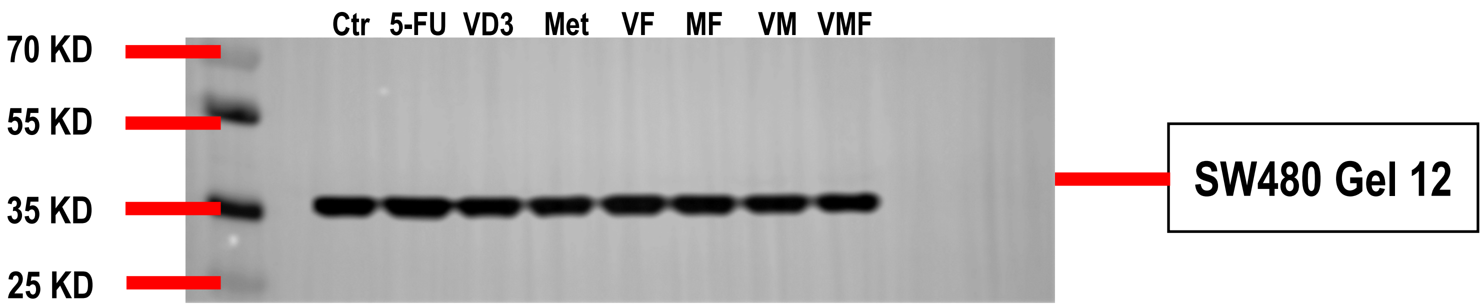

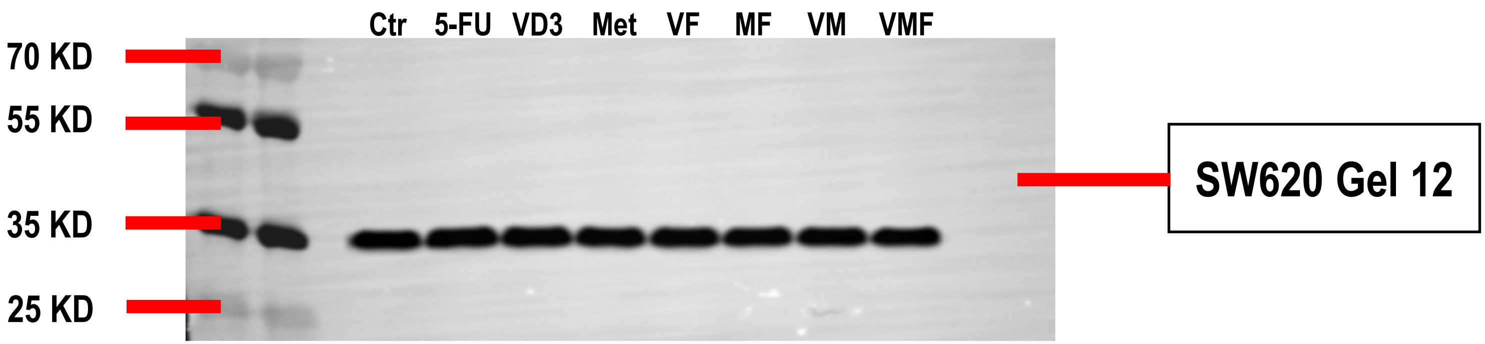

Supplement: Supplementary file 1 [file cancers-14-01538-s001.zip › cancers-1634943-Figure S3.pdf]
