# Supplementary material for: In Vivo and In Vitro Enhanced Tumoricidal Effects of Metformin, Active Vitamin D3, and 5-Fluorouracil Triple Therapy against Colon Cancer by Modulating the PI3K/Akt/PTEN/mTOR Network
Source: Cancers (Basel). 2022 Mar 17;14(6):1538. doi: 10.3390/cancers14061538 (PMC8946120; doi:10.3390/cancers14061538)
Supplement: Supplementary file 1 [file cancers-14-01538-s001.zip › cancers-1634943-Figures S1,S2, Tables S1,S2.pdf]

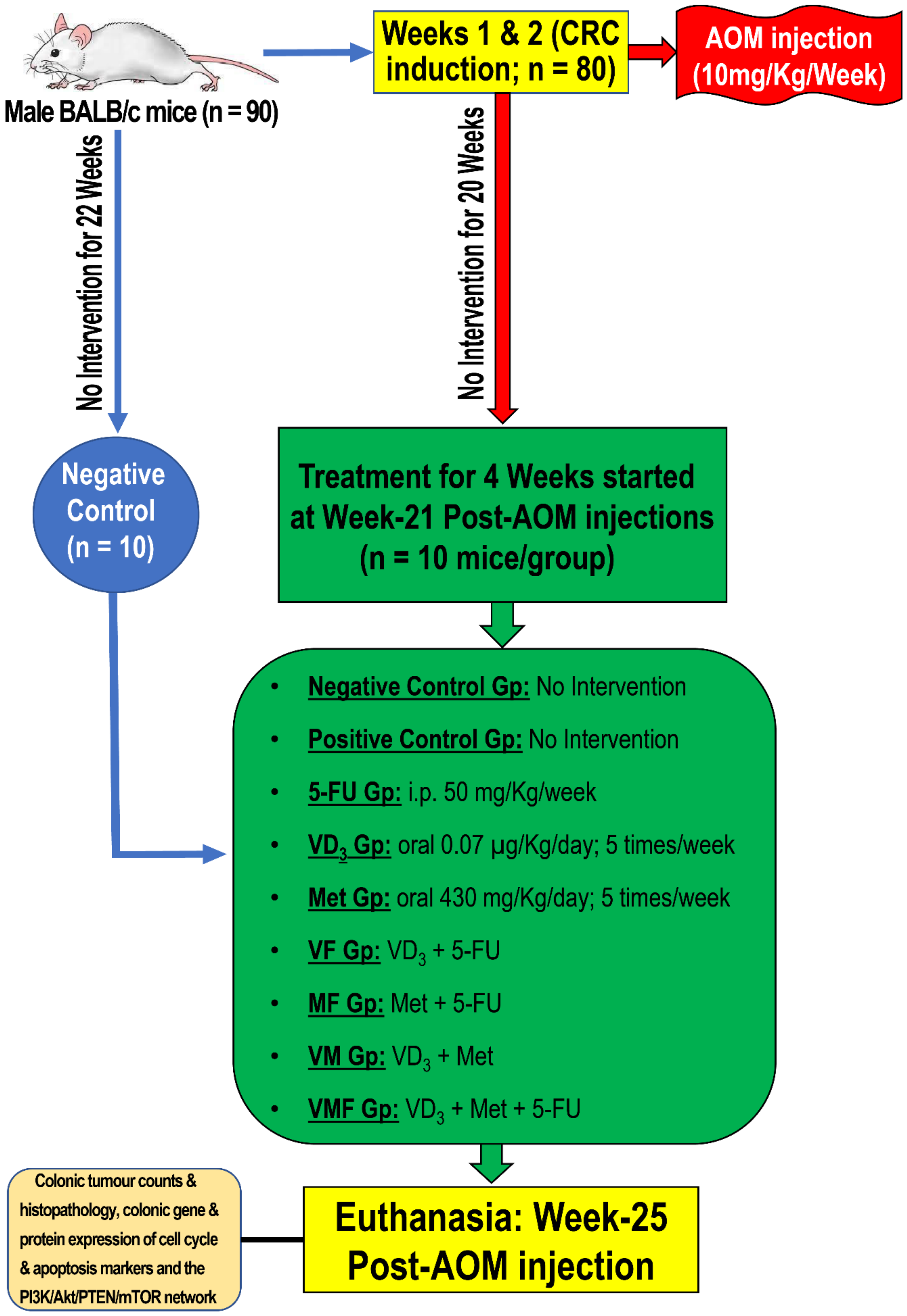

Supplementary Figure S1: Summary of *in vivo* experimental design. A total of 90 male BALB/c mice were used and CRC was induced in 80 animals by azoxymethane (AOM) intraperitoneal injections for two successive (10 mg/Kg/week). Treatments with 5-FU (50 mg/Kg/week), active VD_3 (0.07 µg/Kg/day; five times/week) and/or metformin (430 mg/Kg/day; five times/week) single, dual and triple therapies started at week-21 post AOM injections and lasted for 4 weeks.

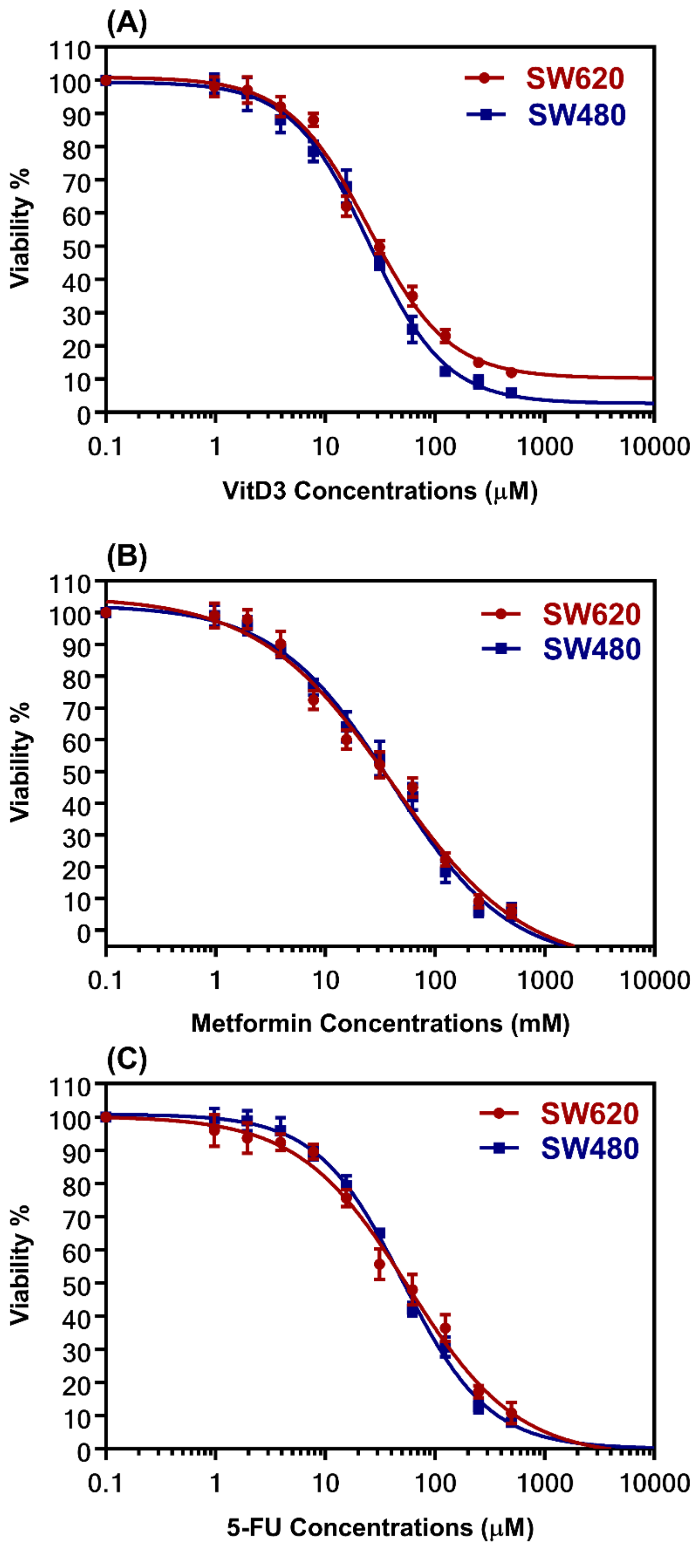

Supplementary Figure S2: Cells were treated with different concentrations of the drugs of interest for 24h, and the IC50 for each determined using the MTT cell viability assay.

Supplementary Table S1. The sequences of PCR primers used for the detection of human *GAPDH*, *PI£KCA, AKT1, mTOR, PTEN, CCND1, CCND3, CDKN1A, CDKN1B, BCL2, Cytochrome C, and Caspase-3* mRNAs in colon samples including the corresponding genes accession numbers and amplicon sizes.

Genes	Forward	Reverse	Amplicon size
GAPDH (NCBI: NM_002046.5)	5' CAC ATG GCC TCC AAG GAG TAA 3'	5' TGA GGG TCT CTC TCT TCC TCT TGT 3'	74 bp
PIK3CA (NCBI: NM_006218.4)	5' GGA CCC GAT GCG GTT AGA G 3'	5' ATC AAG TGG ATG CCC CAC AG 3'	168 bp
AKT1 (NCBI: NM_001382431.1)	5' CTC AGT GTC GTC AGA GCC C 3'	5' ATG GAA AGC AGG CCA GAC TC 3'	100 bp
mTOR (NCBI: NM_004958.4)	5' GAC GAG AGA TCA TCC GCC AG 3'	5' ACA AGG GAC CGC ACC ATA AG 3'	97 bp
PTEN (NCBI: NM_000314.8)	5' CTC AGC CGT TAC CTG TGT GT 3'	5' AGG TTT CCT CTG GTC CTG GT 3'	129 bp
CCND1 (NCBI: NM_053056.2)	5' TGA CCC CGC ACG ATT TCA TT 3'	5' CAT GGA GGG CGG ATT GGA AA 3'	143 bp
CCND3 (NCBI: NM_001136017.3)	5' GGT GCA ATC CTC TCC TCG C 3'	5' TAG TTC ATG GCC AGG GGG AA 3'	183 bp
CDKN1A (NCBI NM_000389.4)	5' AGT CAG TTC CTT GTG GAG CC 3'	5' GCA TGG GTT CTG ACG GAC AT 3'	109 bp
CDKN1B (NCBI: NM_004064.4)	5' CTG GCC TCA GAA GAC GTC AAA 3'	5' AGG ATG TCC ATT CCA TGA AGT CAG 3'	147 bp
BCL2 (NCBI: NM_000633.3)	5' CTT TGA GTT CGG TGG GGT CA 3'	5' GGG CCG TAC AGT TCC ACA AA 3'	162 bp
Cytochrome C (NCBI: NM_018947.6)	5' CGT TGT GCC AGC GAC TAA AA 3'	5' TGG CAC TGG GAA CAC TTC AT 3'	88 bp
BAX (NCBI: NM_001291428.1)	5' TCG CCC TTT TCT ACT TTG CCA 3'	5' GTC CTG GAG ACA GGG ACA TCA 3'	195 bp
CASP3 (NCBI: NM_004346.3)	5' CTC TGG TTT TCG GTG GGT GT 3'	5' CCA CTG AGT TTT CAG TGT TCT CC 3'	90 bp

Supplementary Table S2: Serum concentrations (mean ± SD) of liver enzymes, total protein, albumin, creatinine, and urea in the different study groups.

	NC group	PC group	5-FU group	VD3 group	Met group	VF group	MF group	VMF Group
**ALT (IU/L)**	58.7 ± 5.1	61.02 ± 4.6	63.3 ± 5.1	57.9 ± 6.4	58.3 ± 5.7	57.8 ± 4.7	59.1 ± 4.4	60.2 ± 5.5
**AST (IU/L)**	51.3 ± 6.3	53.2 ± 4.7	56.1 ± 7.2	52.9 ± 5.9	52.5 ± 6.6	54.8 ± 5.6	52.7 ± 6.6	51.8 ± 4.7
**ALP (IU/L)**	108.1 ± 12.9	105.2 ± 10.3	111.4 ± 17.6	107.9 ± 11.2	105.8 ± 10.3	101.5 ± 13.2	107.8 ± 9.9	105 ± 13.3
**Total bilirubin (mg/dL)**	0.45 ± 0.2	0.48 ± 0.3	0.47 ± 0.3	0.45 ± 0.3	0.46 ± 0.1	0.49 ± 0.3	0.46 ± 0.1	0.46 ± 0.09
**Total protein (g/dL)**	7.5 ± 0.6	7.4 ± 0.4	7.1 ± 0.7	7.6 ± 0.5	7.2 ± 0.3	7.4 ± 0.6	7.8 ± 0.7	7.5 ± 0.6
**Albumin (g/dL)**	4.1 ± 0.33	3.7 ± 0.41	4.0 ± 0.45	4.4 ± 0.57	4.2 ± 0.44	3.8 ± 0.41	4.2 ± 0.34	4.3 ± 0.46
**Creatinine (mg/dL)**	0.47 ± 0.08	0.51 ± 0.12	0.48 ± 0.11	0.48 ± 0.14	0.53 ± 0.15	0.46 ± 0.21	0.53 ± 0.24	0.50 ± 0.16
**Urea (mg/dL)**	41.2 ± 6.8	41.3 ± 5.7	43.2 ± 5.4	43.4 ± 6.1	40.4 ± 7.3	42.7 ± 6.6	40.8 ± 6.9	43.3 ± 6.1
